# Acute changes in blood metabolites and amino acid profile post-exercise in Foxhound dogs fed a high endurance formula*

**DOI:** 10.1017/jns.2014.46

**Published:** 2014-09-30

**Authors:** Maria R. C. de Godoy, Alison N. Beloshapka, Rebecca A. Carter, Andrea J. Fascetti, Zengshou Yu, Bridgett J. McIntosh, Kelly S. Swanson, Preston R. Buff

**Affiliations:** 1 Department of Animal Sciences, University of Illinois, Urbana, IL 61801, USA; 2 Division of Nutritional Sciences, University of Illinois, Urbana, IL 61801, USA; 3 The Nutro Company, Franklin, TN 37067, USA; 4 Department of Molecular Biosciences, School of Veterinary Medicine, University of California, Davis, CA 95616, USA; 5 Department of Animal Science, University of Tennessee, Knoxville, TN 37996, USA

**Keywords:** Canine, Exercise, Oxidative stress, Performance, AA, amino acids, BCAA, branched-chain amino acids, BW, body weight, CK, creatine kinase

## Abstract

Dogs participating in endurance exercise, including herding, hunting and racing have a greater energy requirement and may be more susceptible to nutrient depletion, electrolyte imbalance and metabolic stress. The objective of the present study was to investigate the acute response to unstructured mixed exercise in American Foxhounds fed a nutrient-fortified endurance diet. Thirty-nine adult Foxhound dogs (median age: 5·0, range: 2–10 years and median body weight (BW): 36·4, range: 24·9–49·5 kg) were allotted to a standard performance diet (Control) or nutrient-fortified endurance diet for adult dogs (Test). Dogs were balanced by sex, age, BW and athletic performance between diets. All male dogs were intact, whereas all the female dogs were spayed. After 80 d on diet, blood samples were collected via jugular puncture at baseline (0 h), and at 3 and 25 h post-exercise (mean: 17·7 (sem 0·92) km run over 2–3 h). Plasma taurine concentration and complete amino acid (AA) profile, serum chemistry and creatine kinase were measured. Serum chemistry profile remained within normal ranges throughout the study. A significant (*P* < 0·05) diet by time interaction was observed for calcium, alkaline phosphatase and most AA. Plasma taurine and most essential AA were increased (*P* < 0·05) after exercise and remained greater (*P* < 0·05) in dogs fed the Test diet, including the branched-chain AA (isoleucine, leucine and valine). Creatine kinase increased (*P* = 0·01) after 3 h and returned to baseline after 25 h post-exercise, but was not altered by diet. These data indicate that dogs undergoing a moderate bout of exercise did not suffer from electrolyte imbalance, and that a nutrient-fortified diet resulted in greater plasma taurine and essential AA concentrations.

Athlete dogs have a greater energy requirement than adult dogs during periods of normal maintenance^(^^1^^)^. Endurance exercise, characterised by prolonged periods of exercise at submaximal levels of exertion, relies mostly on aerobic metabolism using fat as the primary source of energy. During shorter bouts of greater intensity exercise a shift towards anaerobic metabolism occurs and carbohydrate metabolism becomes important to maintain performance^(^^2^^)^. Electrolyte balance and metabolic stress are important nutritional considerations for canine athletes, as some minerals can become depleted and a greater oxidative rate can affect their performance and health.

Exercise increases the body's production of free radicals and markers of oxidation, while antioxidant concentration depletes them. Therefore, dietary supplementation with antioxidants may have a protective effect against oxidative stress^(^^3^^)^. Dietary protein, including amino acids (AA), are important to minimise striated muscle tissue damage during exercise and thus support athletic performance. The provision of a well-balanced, high-quality diet is necessary to improve endurance and health of athlete dogs. The objective of the present study was to investigate the acute response to unstructured mixed exercise in American Foxhounds fed a nutrient-fortified high endurance diet.

## Material and methods

### Animals, diets and housing

Forty adult Foxhound dogs (thirty-two intact males and seven spayed females) with a median age: 5·0 years (range: 2–10 years) and median body weight (BW): 36·4 kg (range: 24·9–49·5 kg) were used in the present experiment. Power analysis was performed to determine the necessary number of dogs per treatment, using a power of 0·8 and alpha of 0·05. According to values reported by Dunlap *et al.*^(^^4^^)^ for plasma creatine kinase (CK), an sd two times greater than the observed by these authors was assumed for the determination of minimum sample size in the present study. The power analysis indicated a sample size of twenty dogs per treatment. One dog was removed for medical reasons unrelated to the study and its data were removed from statistical analysis. Dogs were group housed throughout the trial, separated by dietary treatment and sex. During the day, all dogs were allowed 8 h in a play yard, which had an approximate dimension of 18 m × 21 m. All dogs had 30 min of human interaction as social enrichment daily as a group. The outdoor runs contained trees for shade, or covered, raised platforms. At night, and in inclement weather, all dogs were housed in a lodge, which allowed access to the outdoors should dogs prefer it. Suspended platforms and beds were available for animals in the lodge area. The indoor facility had natural and artificial lighting and was equipped with fans for comfort if needed. An artificial light:dark cycle of 12 : 12 h was used to allow early morning feeding (05·00 h) and handling the animals until later in the evening (17·00 h). The animal facility and play yard were cleaned daily. The facility and all experimental methods were approved by the Waltham Centre for Pet Nutrition Animal Ethics and Welfare Committee and an informed consent was received by the owner of the kennel (Hard Away Whitworth Hounds) and dogs prior to experimentation. During the experiment, the low and high daily environmental temperature was 3·9 and 24·4°C, respectively.

Animals were assigned to one of two groups; a Control diet (standard commercial performance diet; *n* 19) or a Test diet (nutrient-fortified endurance diet for adult dogs; *n* 20 – NUTRO^®^ NATURAL CHOICE^®^ High Endurance Chicken Meal Whole Brown Rice & Oatmeal Formula). The Test diet provided about 73·6 g of protein, 52·9 g of fat/4814 kJ metabolisable energy (1 kcal = 4·184 kJ), and had a metabolisable energy of 18·7 kJ/g – calculated based on NRC^(^^5^^)^, whereas the Control diet provided 65·7, 51·0 g of protein and fat/4814 kJ and a metabolisable energy of 17·9 kJ/g – calculated based on NRC^(^^5^^)^, respectively. Complete listing of ingredients and nutrient composition of Control and Test diets are presented in Supplementary Table 1 – online. Animals were systematically allotted between groups to match for age, sex, BW and past performance (good, moderate, poor and unknown) to ensure an even distribution across both groups. Dogs were group fed once a day to maintain ideal BW and ration was adjusted as needed during the study between 13 389 and 15480 kJ/dog. Ideal BW was based off previous feeding records, breed standards and BCS using a nine-point scale^(^^6^^)^. Water was available *ad libitum*.

### Blood collection, handling and analyses

Venous blood samples (20 ml) were collected via jugular venipuncture at baseline (0 h), and at 3 and 25 h post-exercise into sodium heparin- or serum-separating tube-vacutainer blood tubes (Becton Dickson). Heparin-vacutainer tubes were immediately placed on ice for approximately 30 min. Heparin and serum tubes were centrifuged at 1240 ***g*** for 10 min at 4°C. Serum, plasma and blood samples were stored at −80°C for plasma AA and MDA analyses, and −20°C for serum chemistry and CK determination. Blood and plasma taurine concentrations, and complete AA profile were analysed within 15 d of blood collection, using a norleucine standard and an automated AA analyser (HPLC, Biochrom 30, Biochrom Ltd) at the Amino Acid Laboratory at the University of California, Davis^(^^7^^)^. Half a millilitre of 6 % sulphosalicylic acid was added to 0·5 ml of plasma to precipitate the plasma proteins. All AA results were reported as nmol/ml of plasma or whole blood. Plasma MDA concentrations were determined within 1 month of blood collection, by MP Biomedicals using commercial kit (SafTest ALDESAFE™, product no. 07KTAC1010) developed based on the method by Hamilton & Rossell^(^^8^^)^. Serum tubes were kept at room temperature. Serum chemistry (creatinine, blood urea nitrogen, total protein, albumin, calcium, potassium, chloride, corticosteroid-induced alkaline phosphatase, alkaline phosphatase, alanine aminotransferase, gamma-glutamyl transpeptidase, total bilirubin, cholesterol, TAG, CO_2_ and glucose) and CK were determined within 1 week of blood collection, using a Hitachi 911 clinical chemistry analyser (Roche Diagnostics; University of Illinois Veterinary Medicine Diagnostics Laboratory).

### Exercise regimen and GPS measurements

The exercise regimen consisted of a bout of 2–3 h of unstructured mixed exercise that simulated a chasing or herding field exercise. Dogs were evenly assigned to two groups by dietary treatment and completed the bout of exercise on one of two consecutive days. All animals were maintained on respective dietary treatments (Control or Test diet) for 80 d and were exercised weekly prior to exercise data collection. Dogs were acclimated to wearing GPS collars (DC 40, Garmin Ltd.) prior to data collection day. Activity data were collected using Astro 320 Global Positioning System (Garmin Ltd.). Because GPS coordinates were provided every 15 s, distance travelled and the time required were obtained and used to calculate total miles run and speeds per dog. The maximum speed was based on the intermittent measurements of distance obtained in a set length of time (e.g. every 15 s). Maximum average speed was considered to be the point of 80 % of maximum speed.

### Statistical analysis

Data from blood metabolites were analysed as repeated measurements using Mixed procedure of SAS (version 9·3, SAS Inst., Inc., Cary, NC). The statistical model included fixed effect of diet, time and their interaction and random effect of animal. The GPS data were analysed using Mixed procedure of SAS. Data normality was checked using the UNIVARIATE procedure of SAS. All treatment least-squares means were compared with each other and Tukey adjustment was used to control for experiment-wise error. Differences between means with *P* < 0·05 were considered significant. Data are presented as means with their standard errors.

## Results

### Blood metabolites and amino acid profile

Serum chemistry profile was within reference range for dogs fed the Control or Test diet (Table 1). A significant diet by time interaction was observed for calcium and alkaline phosphatase concentrations. Serum calcium concentration decreased post-exercise in dogs fed the Control and the Test diet and was lowest after 25 h of exercise for dogs being fed the Test diet (*P* = 0·01). Post acute-exercise, an increased concentration of alkaline phosphatase was observed only in dogs fed the Test (*P* = 0·01). A significant diet effect was noted for blood urea nitrogen (*P* = 0·01), phosphorus (*P* = 0·04), chloride (*P* = 0·02), cholesterol (*P* = 0·01) and CO_2_ (*P* = 0·01) serum concentrations. Similarly, most of the serum metabolites were affected by time, except for alanine aminotransferase (*P* = 0·07), gamma-glutamyl transpeptidase (*P* = 0·33) and MDA (*P* = 0·77). Among the variables affected by time, serum CK, an indirect marker of muscle damage, was sharply increased 3 h post-exercise (*P* = 0·01), but it remained within physiological range and returned to baseline concentration at 25 h post-exercise. Plasma MDA, an indirect marker of oxidative stress and lipid peroxidation, did not differ prior to or post-exercise in dogs fed Control or Test diet (*P* = 0·89; Table 1).

**Table 1.** Serum chemistry, creatine kinase and malondialdehyde concentrations prior to and 3 or 25 h post-exercise in American Foxhound dogs fed a Control or Test diet

		Treatments									
		Control			Test				*P*		
Item*	Reference range	0 h	3 h	25 h	0 h	3 h	25 h	sem	Diet	Time	D × T
CRE (µmol/l)	44·2–132·6	88·2	79·6	85·7	81·3	84·0	91·1	0·03	0·38	0·01	0·21
BUN (mm/l)	2·1–10·7	6·8	5·9	7·3	7·1	6·5	8·4	0·71	0·01	0·01	0·08
TP (g/l)	51·0–70·0	68·6	70·6	68·8	70·6	71·2	69·3	0·13	0·55	0·01	0·21
ALB (g/l)	0·03–0·04	0·03	0·03	0·03	0·03	0·03	0·03	0·06	0·79	0·01	0·36
Ca (mm/l)	1·9–2·9	2·6^a^	2·5^b^	2·5^b^	2·5^a^	2·5^b^	2·4^c^	0·04	0·46	0·01	0·01
P (mm/l)	0·9–1·7	1·5	1·5	1·3	1·6	1·5	1·5	0·06	0·04	0·01	0·11
Na (mm/l)	141–152	144·21	143·89	145·32	144·55	143·75	145·15	0·48	0·98	0·01	0·81
K (mm/l)	3·9–5·5	4·3	4·0	4·2	4·3	4·2	4·4	0·06	0·01	0·01	0·10
Cl (mm/l)	107–118	109·84	106·58	109·47	110·45	109·65	111·45	0·73	0·02	0·01	0·08
CIALP (U/l)		1·63^c^	1·37^c^	1·90^c^	2·9^bc^	3·60^b^	4·25^a^	0·91	0·13	0·01	0·01
ALP (U/l)	7–92	33·42^c^	35·32^c^	39·68^c^	44·50^c^	49·65^b^	60·60^a^	3·67	0·01	0·01	0·01
ALT (U/l)	8–65	34·90	37·05	33·79	37·05	46·45	41·35	3·42	0·09	0·07	0·39
GGT (U/l)	0–7	1·63	1·37	1·68	1·25	1·30	1·45	0·19	0·25	0·33	0·61
TBIL (µmol/l)	1·7–5·1	2·2	2·9	2·6	2·2	2·7	2·4	0·17	0·55	0·01	0·79
CHOL (mm/l)	3·3–7·7	4·4	4·3	4·5	3·9	3·8	3·9	0·16	0·01	0·01	0·47
TAG, mm/l	0·4–1·7	0·6^bc^	0·5^c^	0·8^ab^	0·5^c^	0·6^c^	1·0^a^	0·06	0·61	0·01	0·03
CO_2_, mm/l	16–24	27·05	25·63	24·11	24·60	23·55	21·00	0·42	0·01	0·01	0·45
GLUCOSE (mm/l)	3·8–7·0	5·0	5·9	5·3	4·9	5·5	5·1	0·12	0·05	0·01	0·58
CK (U/l)	26–791	168·42	490·42	142·63	149·00	445·35	115·25	44·17	0·39	0·01	0·94
MDA (µmol/l)		7·37	6·71	6·96	6·97	6·83	6·87	0·55	0·80	0·77	0·89

^a–c^ Within a row, means without a common letter differ (diet × time interaction; *P* < 0·05), *n* 39.

*Creatinine = CRE, blood urea nitrogen = BUN, total protein = TP, albumin = ALB, calcium = Ca, phosphorus = P, potassium = K, chloride = Cl, corticosteroid-induced alkaline phosphatase = CIALP, alkaline phosphatase = ALP, alanine aminotransferase = ALT, gamma-glutamyl transpeptidase = GGT, total bilirubin = TBIL, cholesterol = CHOL, carbon dioxide = CO_2_, creatine kinase = CK, malondialdehyde = MDA.

A significant diet by time interaction was observed for most of the AA analysed, except for arginine (*P* = 0·52), phenylalanine (*P* = 0·16), aspartic acid (*P* = 0·30), glutamic acid (*P* = 0·87), glutamine (*P* = 0·14), hydroxyproline (*P* = 0·82), amino butyric-acid (*P* = 0·08), cystathionine (*P* = 0·67) and 2-methyl-l-histidine (*P* = 0·22; Table 2). In general, essential AA plasma concentrations were increased after exercise and in animals fed the Test diet; isoleucine (*P* = 0·01), leucine (*P* = 0·01), lysine (*P* = 0·01), methionine (*P* = 0·01), threonine (*P* = 0·01) and valine (*P* = 0·01); in comparison with AA concentrations prior to exercise and/or from animals fed the Control diet. A similar pattern was observed for some non-essential AA. Plasma taurine concentration was greater (*P* = 0·01) prior to and post-exercise in dogs fed the Test diet in comparison with AA concentrations prior to exercise and/or from animals fed the Control diet. Plasma carnosine also was increased (*P* = 0·01) in dogs fed the Test diet at 25 h post-exercise when compared with dogs fed the Control diet.

**Table 2.** Amino acid profile prior to and 3 or 25 h post-exercise of American Foxhound dogs fed a Control or Test diet

	Treatments									
	Control			Test				*P*		
AA (nmol/ml)	0 h	3 h	25 h	0 h	3 h	25 h	sem	Diet	Time	D × T
WB Taurine	379·6^c^	431·1^c^	353·0^b^	475·0^b^	608·5^a^	482·7^b^	19·69	0·01	0·01	0·01
Plasma Taurine	61·8^c^	78·8^c^	73·9^c^	152·1^b^	229·6^a^	173·3^b^	9·07	0·01	0·01	0·01
*Essential*										
Arginine	137·6	147·9	137·4	155·9	175·0	168·7	6·31	0·01	0·06	0·52
Histidine	82·2^bc^	96·8^a^	80·2^c^	85·1^bc^	98·1^a^	92·1^ab^	2·62	0·06	0·01	0·01
Isoleucine	56·8^c^	71·5^b^	61·9^bc^	70·8^b^	102·9^a^	94·4^a^	2·70	0·01	0·01	0·01
Leucine	132·2^c^	155·7^b^	146·6^bc^	134·3^c^	190·6^a^	178·4^a^	4·46	0·01	0·01	0·01
Lysine	104·0^c^	159·4^b^	116·0^c^	145·1^b^	208·6^a^	191·6^a^	6·51	0·01	0·01	0·01
Methionine	81·5^b^	74·2^b^	84·5^b^	77·0^b^	85·2^b^	103·9^a^	3·28	0·01	0·01	0·01
Phenylalanine	54·1	63·8	56·6	52·9	71·1	58·4	2·30	0·24	0·01	0·16
Threonine	243·6^b^	229·0^b^	227·2^b^	252·5^b^	242·7^b^	302·0^a^	13·12	0·05	0·01	0·01
Tryptophan	70·7^b^	93·2^a^	82·3^a^	66·1^b^	96·8^a^	96·3^a^	3·88	0·31	0·01	0·01
Valine	179·1^d^	228·4^b^	215·0^bc^	198·7^cd^	270·6^a^	275·1^a^	6·71	0·01	0·01	0·01
*Non-essential*										
Alanine	362·2^b^	478·0^a^	356·9^b^	370·7^b^	475·1^a^	430·2^a^	17·48	0·16	0·01	0·01
Aspartic acid	3·5	6·0	7·7	4·9	6·6	8·9	0·29	0·01	0·01	0·30
Asparagine	59·6^d^	74·6^c^	68·8^c^	78·2^c^	98·2^b^	110·7^a^	3·69	0·01	0·01	0·01
Glutamic acid	25·6	25·9	25·1	27·9	27·6	27·7	1·03	0·05	0·90	0·87
Glutamine	626·1	733·9	619·0	533·0	691·9	599·3	19·03	0·01	0·01	0·14
Glycine	383·1	392·0	460·3	276·7	329·6	429·7	18·02	0·01	0·01	0·05
Hydroxylysine	9·7^b^	16·4^a^	10·5^b^	10·9^b^	12·1^ab^	14·0^ab^	1·26	0·89	0·01	0·01
Hydroxyproline	109·8	74·9	111·0	109·0	64·2	110·3	9·67	0·64	0·01	0·82
Proline	217·6^c^	231·8^bc^	266·3^ab^	168·0^d^	226·2^bc^	285·0^a^	11·85	0·27	0·01	0·01
Serine	179·7^a^	146·0^c^	168·7^ab^	166·5^bc^	147·3^bc^	194·2^a^	7·36	0·58	0·01	0·01
Tyrosine	54·9	51·0	48·7	44·5	48·6	45·6	1·89	0·01	0·19	0·06
*Others*										
Amino-butyric acid	15·4	29·9	17·1	25·9	33·1	25·2	1·71	0·01	0·01	0·08
Carnosine	39·7^b^	41·0^b^	40·2^b^	38·8^b^	39·7^b^	47·9^a^	1·56	0·27	0·02	0·01
Citruline	58·5	52·5	54·1	50·0	46·5	52·7	3·03	0·18	0·01	0·05
Cystathionine	4·2	3·6	3·9	4·0	3·1	3·9	0·31	0·46	0·04	0·67
Ethanolamine	15·9^c^	23·2^a^	14·9^c^	21·2^ab^	15·5^c^	17·9^bc^	1·26	0·87	0·06	0·01
1-Methyl-l-histidine	14·6^c^	13·1^c^	16·0^c^	25·0^b^	27·4^b^	35·2^a^	1·58	0·01	0·01	0·01
2-Methyl-l-histidine	16·1	16·3	16·3	13·7	17·4	16·5	1·55	0·86	0·15	0·22
Ornithine	16·5^bc^	23·0^a^	17·7^bc^	13·7^c^	25·6^a^	21·4^ab^	1·20	0·31	0·01	0·01

^a–c^Within a row, means without a common letter differ (diet × time interaction; *P* < 0·05), *n* 39.

A significant diet by time (D × T) interaction was observed for plasma branched-chain amino acid (BCAA) and tryptophan concentrations (*P* = 0·01; Table 2). For the BCAA:tryptophan ratio data, there was only a significant effect of diet, with the Test diet having a greater (*P* = 0·02) ratio when compared with the Control diet (6·1 ± 0·24 *v.* 5·3 ± 0·25).

### Athletic performance

Distance run (km), average speed (km/h) and maximum speed (km/h) during the exercise bout did not differ (*P* = 0·48, 0·84 and 0·79, respectively) between dogs fed the Control (17·1(0·91) km; 9(0·37) km/h; 36·3 (2·0) km/h, respectively) and Test (18·6(0·93) km; 9(0·37) km/h; 37·1(2·0) km/h, respectively) diets.

## Discussion

The present study examined the acute response to unstructured mixed exercise in American Foxhounds fed a nutrient-fortified endurance diet on blood metabolites, plasma indirect oxidative marker and athletic performance. Serum chemistry results indicated that all dogs were healthy prior and post-exercise. Even though some metabolites changed due to exercise, diet or their combination, they were still within reference range of healthy animals. Increased plasma or serum concentration of CK following physical activity has been associated with muscle damage that can result in soreness, decreased range of motion, swelling and performance decline^(^^9^^)^. In human athletes, elevated plasma CK concentration up to 5 d post-exercise has been observed^(^^9^^)^. In sled dogs, plasma CK activity was significantly increased post-exercise and incremental increase in CK was observed from day 1 (519 U/l) of exercise when compared with days 2 and 3 (2332 and 2487 U/l, respectively)^(^^3^^)^. In that study, a group of dogs received daily antioxidant supplementation (457 mg of vitamin E, 706 mg of vitamin C and 5·1 mg of β-carotene), however antioxidant supplementation failed to attenuate an exercise-induced increase in CK activity^(^^3^^)^. This outcome is in agreement with the present study, where no beneficial effect in plasma CK attenuation was observed with the provision of a nutrient-fortified diet for athlete dogs.

Differently than hypothesised, plasma MDA concentration was not affected by diet or exercise. Even though aerobic exercise leads to a greater consumption of oxygen, and as a consequence an increase in free radical production^(^^3^^)^, the Control and the Test diet must have provided sufficient antioxidants that protected these animals from oxidative stress. Another possibility is that the exercise regimen was not severe enough to challenge the animals' oxidative rate. Previous research has reported plasma MDA concentration for adult healthy dogs to range from 5 to 11 µmol/l^(^^10^^)^. Therefore, in the present study, dogs from Control and Test diets pre- and post-exercise were within the range reported for healthy dogs.

In the present study, the greater plasma and blood taurine concentrations of dogs fed the Test diet was likely a consequence of increased dietary taurine content. Taurine is important for maintenance of cardiac function, which is fundamental for maintenance of animals' health and athletic performance^(^^7^^)^. In the present study, all dogs had plasma and whole blood taurine concentrations above low critical values (concentrations considered near to cause a nutrient deficiency) reported in the literature for adult healthy dogs (40 and 150 nmol/ml, respectively)^(^^7^^)^. However, three dogs from the Control group had plasma taurine concentration close to the minimal critical value (40 nmol/ml). Overtime and with continuous exercise, these animals could be at greater risk to develop taurine deficiency, suggesting that canine athletes might conditionally require dietary taurine; however, this hypothesis requires further investigation.

Increased concentration of plasma carnosine has a potentially ergogenic effect; enhancing buffering capacity in skeletal muscle^(^^11^^)^. In addition, racing dogs (Greyhounds) have greater intramuscular carnosine concentration^(^^12^^)^, probably a protective mechanism to avoid exercise-induced acidosis during anaerobic exercise. The increased plasma carnosine concentration observed at 25 h post-exercise in dogs fed the Test diet was not associated with improved athletic performance in the present study, however.

Maintenance of greater plasma BCAA concentrations post-exercise may aid animals to recover faster, because these AA are involved in protein synthesis, energy supply for skeletal muscle and suppression of lactate concentration in muscle during exercise^(^^13^^–^^15^^)^. Increased BCAA:tryptophan ratio has been previously related to delay mental and physical fatigue during sustained heavy exercise^(^^16^^,^^17^^)^. Among the BCAA, leucine is primarily involved with protein synthesis^(^^18^^)^. In the present study, plasma leucine concentration was increased post-exercise in dogs fed the Test diet. However, no differences in exercise performance (distance run, and average and maximum speed) were observed between dogs fed the Control *v.* the Test diet after a bout of unstructured mixed exercise. Future studies are warranted to investigate the effects of more strenuous exercise regimens on the plasma BCAA concentrations of exercising dogs and their potential benefits in delaying fatigue and mitigating muscle damage. Despite some of the benefits of feeding the Test diet (e.g. increased plasma taurine, carnosine and BCAA concentrations) no improvement in athletic performance was observed between dogs fed the Control or Test diets, which indicates that both diets were adequate to maintain athletic performance and health of dogs undergoing unstructured mixed exercise.

Some of the limitations associated with this field study are that not all parameters investigated could be as tightly controlled as in a research facility. In the present study, some of the limitations included the inability to measure individual food intake, which limits the data interpretation because the effect of diet cannot be separated from hierarchical dominance during feeding. In addition, the unstructured exercise regimen may not have challenged all animals in the same manner. A structured, longer and more intense exercise regimen could have been beneficial; however it would not reflect the free-range exercise of a hunting dog. Variations in age and athletic performance might also have restricted our ability to detect differences due to exercise and diet, even though animals were balanced for these variables. Despite these limitations, the data presented herein reflects variations commonly faced in field research studies and in the pet exercising population. Another limitation of the present study is that, to our knowledge, this was the first research study to use a GPS device to provide performance data. This GPS device was developed to accurately track the location of hunting dogs and has been used in this regard for years, but has not been validated for use in a research setting. Because the device provides GPS-based location data every 15 s (including the initial location of the animal, the distance travelled and the amount of time needed to travel the distance), we were able to calculate distance run, and average and maximum speed during exercise bout.

### 

#### Conclusions

In summary, feeding a nutrient-fortified diet resulted in greater plasma taurine concentration pre- and post-acute-exercise, and greater plasma BCAA:tryptophan post-exercise. While the latter has been related to delayed fatigue in response to exercise, the greater BCAA:tryptophan ratio in the present study did not affect athletic performance. In addition, no effect of diet was observed on the athletic performance. Similarly, plasma MDA concentration was not affected by exercise or dietary treatment. It is possible the exercise regimen used in the present experiment was not strenuous enough to pose a metabolic and oxidative challenge for the animals. However, future studies are warranted to investigate the effect of a nutrient-fortified diet in dogs undergoing repetitive exercise or a more vigorous and intense exercise regimen as they may impose a greater oxidative and metabolic stress to athlete dogs.

## Supplementary material

The supplementary material for this article can be found at http://www.journals.cambridge.org/jns
